# Patients’ administration preferences: progesterone vaginal insert (Endometrin®) compared to intramuscular progesterone for Luteal phase support

**DOI:** 10.1186/1742-4755-11-78

**Published:** 2014-11-11

**Authors:** Angeline N Beltsos, Mark D Sanchez, Kevin J Doody, Mark R Bush, Alice D Domar, Michael G Collins

**Affiliations:** Fertility Centers of Illinois, River North Center, 900 N Kingsbury, Ste RW6, Chicago, IL 60610 USA; Women’s Medical Research Group, LLC, Florida Fertility Institute, 2454 McMullen Booth Rd Ste 601, Clearwater, FL 33759 USA; The Center for Assisted Reproduction, 1701 Park Place Ave, Bedford, TX 76022 USA; Conceptions Reproductive Associates of Colorado, 271 W County Line Rd, Littleton, CO 80129 USA; Domar Center for Mind/Body Health, 130 Second Avenue, Waltham, MA 02451 USA; Ferring Pharmaceuticals, Inc, 4 Gatehall Drive, Third Floor, Parsippany, NJ 07054 USA

**Keywords:** Tolerability, Vaginal progesterone, Luteal support, Endometrium, IVF, Polycystic ovary syndrome

## Abstract

**Background:**

Administration of exogenous progesterone for luteal phase support has become a standard of practice. Intramuscular (IM) injections of progesterone in oil (PIO) and vaginal administration of progesterone are the primary routes of administration. This report describes the administration preferences expressed by women with infertility that were given progesterone vaginal insert (PVI) or progesterone in oil injections (PIO) for luteal phase support during fresh IVF cycles.

**Methods:**

A questionnaire to assess the tolerability, convenience, and ease of administration of PVI and PIO given for luteal phase support was completed by infertile women diagnosed with PCOS and planning to undergo IVF. The women participated in an open-label study of highly purified human menopausal gonadotropins (HP-hMG) compared with recombinant FSH (rFSH) given for stimulation of ovulation.

**Results:**

Most women commented on the convenience and ease of administration of PVI, while a majority of women who administered IM PIO described experiencing pain. In addition, their partners often indicated that they had experienced at least some anxiety regarding the administration of PIO. The most distinguishing difference between PVI and PIO in this study was the overall patient preference for PVI. Despite the need to administer PVI either twice a day or three times a day, 82.6% of the patients in the PVI group found it “very” or “somewhat convenient” compared with 44.9% of women in the PIO group.

**Conclusions:**

The results of this comprehensive, prospective patient survey, along with findings from other similar reports, suggest that PVI provides an easy-to-use and convenient method for providing the necessary luteal phase support for IVF cycles without the pain and inconvenience of daily IM PIO. Moreover, ongoing pregnancy rates with the well-tolerated PVI were as good as the pregnancy rates with PIO.

**Trial registration:**

ClinicalTrial.gov, NCT00805935

**Electronic supplementary material:**

The online version of this article (doi:10.1186/1742-4755-11-78) contains supplementary material, which is available to authorized users.

## Background

Progesterone produced by the corpus luteum during the luteal phase of the menstrual cycle promotes secretory transformation of endometrial tissue, which is essential for embryo implantation [1]. Use of gonadotropin-releasing hormone (GnRH) agonists and antagonists administered to patients during cycles of IVF may adversely affect luteal function and compromise the production of progesterone by the corpus luteum [1, 2]. Administration of exogenous progesterone for luteal phase support has become a standard of practice, and its administration has been shown to improve pregnancy rates during cycles of IVF [3–5].

Progesterone is administered by oral, intramuscular (IM), or vaginal routes [3–5]. However, because of poor bioavailability of progesterone administered orally, IM injections of progesterone in oil (PIO) and vaginal administration of progesterone are the primary routes used in clinical practice [6].

Administration of progesterone by IM and vaginal routes appears to result in similar pregnancy rates [3, 6–16]. Notably, a recent retrospective study of a large cohort of women undergoing IVF showed significantly better clinical pregnancy rates when progesterone was given by both the IM and vaginal routes compared with the vaginal route alone for IVF cycles with frozen embryo transfer (*P* < 0.001); but no difference for IVF cycles with fresh autologous (*P* = 0.05) or donor oocytes (*P* = 0.39) [17].

Surveys associated with clinical trials have shown that vaginal administration is preferred over IM administration by most women [15, 18, 19]. Women prefer vaginal over IM administration because vaginal administration is convenient, easy to use, and does not involve the pain and side effects commonly associated with daily IM injections [15, 18, 19].

This report describes the administration preferences expressed by women with polycystic ovarian syndrome (PCOS) who were given progesterone vaginal insert (PVI, Endometrin®, Ferring Pharmaceuticals, Inc, Parsippany, NJ, USA) or IM PIO (Progesterone Injection USP 50 mg/mL, American Reagent, Corp, Shirley, NY, USA) for luteal phase support during fresh IVF cycles.

## Methods

### Patients

To be eligible for this phase 4 study, participants had to be premenopausal (women aged 18–42 years) with a documented history of infertility (e.g., unable to conceive for at least 1 year, or for 6 months for women aged 38 years or older, or with bilateral tubal occlusion or absence of tubal segments, or male factor) previously diagnosed with PCOS and considered to be a favorable candidate to undergo an assisted reproductive technology (ART) procedure. PCOS was defined using the criteria adopted at the 2003 Rotterdam PCOS Consensus [20].

For study inclusion, women were required to have a body mass index (BMI) of 18–39 kg/m^2^; an intact uterus adnexa visualized on transvaginal ultrasonography (TVU) performed within 1 year of screening; and 2 of the following 3 conditions present: (1) oligo- or anovulation, (2) clinical and/or biochemical signs of hyperandrogenism, and/or (3) polycystic ovaries. Women were also required to have an early follicular phase (day 3) serum follicle-stimulating hormone (FSH) concentration ≤ 15 IU/L, and serum estradiol (E_2_) concentration within normal limits for 4 months before screening.

Women were ineligible to participate if they had 2 or more previous failed IVF cycles or poor response to gonadotropins (i.e., development of ≤ 2 mature follicles); planned to use a gestational or surrogate carrier or donor oocytes; had a history of recurrent pregnancy loss (>2); currently had abnormal uterine bleeding of an undetermined origin; or had a male partner with severe male factor infertility who required invasive or surgical sperm retrieval (e.g., microsurgical epididymal sperm aspiration, testicular sperm extraction).

Women were also ineligible if they were diagnosed with clinically relevant systemic disease (e.g., uncontrolled thyroid and adrenal dysfunction, an organic intracranial lesion such as a pituitary tumor, insulin-dependent diabetes mellitus, uterine cancer); had a current or a recent history of substance abuse, including alcohol abuse; smoked 10 or more cigarettes per day; had a hypersensitivity to any of the study drugs; or had any surgical or medical condition that, in the investigator’s or sponsor’s judgment, would interfere with absorption, distribution, metabolism, or excretion of the study drugs.

The study was conducted in accordance with the ethical principles that have their origin in the Declaration of Helsinki, followed the approved protocol, observed International Conference on Harmonization–Good Clinical Practice guidelines, and complied with all applicable regulatory requirements. The protocol and its associated Informed Consent Agreement were reviewed and approved by the appropriate Institutional Review Board (Independent Investigational Review Board, Inc., Women and Infants Hospital of Rhode Island Institutional Review Board and Weill Cornell Medical College Institutional Review Board). Study personnel obtained written informed consent directly from all participants before their entry into the study. This trial is registered under NCT00805935 [https://clinicaltrials.gov/].

### Study design

This multicenter, randomized, open-label, phase 4, exploratory, study was conducted to assess the efficacy and safety of highly purified human menopausal gonadotropins (HP-hMG, Menopur® Ferring Pharmaceuticals, Inc, Parsippany, NJ, USA) compared with recombinant FSH (rFSH) during fresh cycles of IVF in otherwise healthy infertile women who were diagnosed with PCOS and planning to undergo IVF. Women were also randomized to PVI vs PIO. Women who participated in the study completed a questionnaire to assess the tolerability, convenience, and ease of administration of PVI and PIO given for luteal phase support.

Women were screened based on the study’s inclusion/exclusion criteria, analysis of medical and infertility history, evaluation of FSH and E_2_ serum concentrations, results from physical and gynecological examinations, and findings from the center’s standard screening evaluations for IVF participants. TVU was performed and serum was collected for E_2_ and progesterone concentration measurement from all participants at baseline and periodically throughout the study. All adverse events (AEs) and concomitant medications taken by participants were documented throughout the study. Patients returned to the study center for regularly scheduled clinic visits as required per the IVF protocol and at other specified time periods.

### Study treatments

Leuprolide acetate, a GnRH agonist (GnRHa), was given for pituitary downregulation. Participants were allowed to use oral contraceptives before administration of the GnRHa, if this was standard practice at the center. Patients received daily injections of either HP-hMG (Menopur®, Ferring Pharmaceuticals, Inc, Parsippany, NJ, USA) 225 IU (3 vials) subcutaneously (SC) or follitropin beta for injection (rFSH: Follistim®, Merck & Co Inc, Whitehouse Station, NJ, USA) 225 IU SC for a minimum of 5 days for ovarian stimulation. Human chorionic gonadotropin (hCG; Novarel®, Ferring Pharmaceuticals, Inc) was administered to trigger ovulation.

### Luteal phase support

Progesterone was administered to support the luteal phase beginning on the day after oocyte retrieval. Patients were randomized to receive PVI 100 mg inserted vaginally 2 or 3 times daily, or PIO 50-mg IM injection once daily until 10-weeks’ gestation or confirmation of a negative pregnancy test. A large, randomized control trial previously demonstrated that there is no significant difference in pregnancy outcomes between 2 and 3 times daily dosing of PVI [21]. Each patient also took estradiol tablets (Estrace®, Barr Laboratories, Inc, Pomona, NY, USA) 2 mg by mouth once daily for the duration of luteal support. Embryo transfer occurred on day 3 or day 5 after insemination or intracytoplasmic sperm injection.

### Assessments

Patients completed a survey to assess administration preferences of PVI and PIO at the start of their luteal phase support and at their final visit. Questions included on the PVI and PIO patient surveys are shown in the Assessments subsection under Methods. Responses to the survey assessed convenience and ease of use and other variables such as pain and discomfort associated with study drug administration. Assessment of safety and tolerability included documentation of AEs and serious AEs, results from clinical laboratory evaluations and electrocardiograms, measurements of vital signs, and findings from TVU.

### Patient survey assessing administration preferences of progesterone vaginal insert (PVI) and progesterone in oil (PIO)

#### Section 1*† (PVI and PIO)

For the following questions, please choose the response which best reflects your experience.

1. How would you describe the convenience of administering/dosing [Endometrin /progesterone in oil injections]?
  Very convenient
  Somewhat convenient
  Neither convenient or inconvenient
  Somewhat inconvenient
  Very inconvenient
2. How would you describe the ease of administering/dosing [Endometrin/progesterone in oil injections]?
  Very easy
  Somewhat easy
  Neither easy or difficult
  Somewhat difficult
  Very difficult
3. In a previous ART cycle, were you prescribed progesterone supplementation?
  Yes
  No (Skip questions 4, 5).
4. If you answered ‘Yes’ to question 3: Which of the following progesterones were you prescribed in a previous cycle?
  Progesterone in oil
  Crinone/Prochieve
  Prometrium orally
  Prometrium vaginally
  Pharmacy compounded suppositories
  Endometrin
5. If you answered ‘Yes’ to question 3: Overall, how would you compare [Endometrin/ progesterone in oil injections] to the different progesterone used in a previous cycle?
  Very convenient and easy to use
  Somewhat convenient and easy to use
  Neither convenient or inconvenient and easy to use
  Very inconvenient and difficult to use
6. Please rate your overall satisfaction level with [Endometrin/ progesterone in oil injections]
  Very satisfied
  Somewhat satisfied
  Neither satisfied nor dissatisfied
  Somewhat dissatisfied
  Very dissatisfied
7. If given a choice, which progesterone would you prefer to use?
  Progesterone in oil
  Crinone/Prochieve
  Prometrium orally
  Prometrium vaginally
  Pharmacy compounded suppositories
  Endometrin

#### Section 2¶ (PIO only)

1. How painful were the progesterone in oil injections?
  Very painful
  Somewhat painful
  Neither painful nor painless
  Somewhat painless
  Very painless
2. How would you describe your partner’s level of anxiety in giving the progesterone in oil injections?.
  Very anxious
  Somewhat anxious
  Neither anxious nor comfortable
  Not anxious
3. Did the progesterone in oil injections make you consider dropping out of treatment?
  Yes
  No

^*^ Separate surveys were administered for PVI and PIO.

^†^ “Endometrin” or “progesterone in oil injection” was included in the questions on each survey, and referred to PVI or PIO, respectively.

^‡^ Sections 1 and 2 on PVI survey.

^¶^ Section 2 PIO survey.

### Statistical analysis

Fisher’s exact test was used to determine treatment group comparisons for categorical variables. As appropriate, one-way analysis of variance or the Wilcoxon rank sum test were used, to determine treatment group comparisons for continuous variables. Statistical significance was declared if the two-sided *P* value was ≤ 0.05. Statistical analyses were performed using statistical software (SAS version 9.2; SAS Institute Inc, Cary, NC, USA).

## Results

Demographic characteristics were similar for the PVI and PIO treatment groups (Table 1). The majority of women enrolled in the study were Caucasian, with 71.7% and 75.4% in the PVI and PIO groups, respectively. Mean age was 30.9 and 31.5 years in the PVI and PIO groups, respectively.

**Table 1:** **Patient demographics and baseline characteristics, ITT population**

Parameter	PVI	PIO
	(n = 53)	(n = 57)
Age, y	30.9 (20–40)	31.5 (22–41)
BMI, kg/m^2^	26.9 (5.2)	28.9 (6.5)
Race		
White	38 (71.7)	43 (75.4)
Black	0	1 (1.8)
Asian	9 (17.0)	2 (3.5)
Hispanic	6 (11.3)	10 (17.5)
Other	0	1 (1.8)
Duration of infertility, months	32.2 (19.7)	49.6 (44.6)
	26.0 (10–96)	34.0 (12–240)
ART History		
Number previous IVF cycles	0.2 (0.5)	0.2 (0.4)
Number failed IVF cycles	0.1 (0.3)	0.1 (0.3)
Number previous ovulation induction cycles*	4.2 (3.1)	5.3 (6.8)
Number failed ovulation induction cycles^†^	4.1 (3.1)	5.2 (6.8)
Gonadotropin for ovulation induction		
rFSH	28 (52.8)	30 (52.6)
HP-hMG	25 (47.2)	27 (47.4)

Values are *n* (%), median (range), or means (standard deviation).

*Excluding IVF, gamete intrafallopian transfer, and/or pronuclear stage embryo transfer.

^†^Excluding IVF.

ITT, intent to treat; PVI, progesterone vaginal insert; PIO, progesterone in oil; BMI, body mass index; ART, assisted reproductive technologies; rFSH, recombinant follicle-stimulating hormone; HP-hMG, highly purified human menopausal gonadotropin.

The number of women who received PVI or PIO for luteal phase support, underwent embryo transfer, and completed 10 weeks of luteal phase support are shown in Table 2. A negative result on serum pregnancy test was the most frequent reason for discontinuing luteal phase support in both groups (PVI, 26%; PIO, 21%). PVI was administered twice daily in 23.1% and 3 times daily in 76.9% of women randomized to PVI.

**Table 2:** **Disposition of women randomized to luteal support with progesterone vaginal insert and progesterone in oil**

Parameter	PVI	PIO
	(n = 53)	(n = 57)
Received luteal phase support	48 (90.6)	50 (87.7)
Underwent embryo transfer	46 (86.8)	49 (86.0)
Biochemical pregnancy*	32 (60.4)	37 (64.9)
Clinical pregnancy^†^	27 (50.9)	29 (50.9)
Ongoing pregnancy^‡^	25 (47.2)	28 (49.1)

Values are *n* (%).

*Positive result on serum β-hCG test 12–14 days after embryo transfer, *P* = 0.853.

^†^Gestational sac visualized using transvaginal ultrasonography approximately 4 weeks after embryo transfer, *P* = 1.0.

^‡^Fetal heart movements identified using transvaginal ultrasonography at approximately 6 weeks’ gestation, *P* = 0.851.

PVI, progesterone vaginal insert; PIO, progesterone in oil; β-hCG, beta human chorionic gonadotropin.

### Patient-reported outcomes

**Figure 1:**
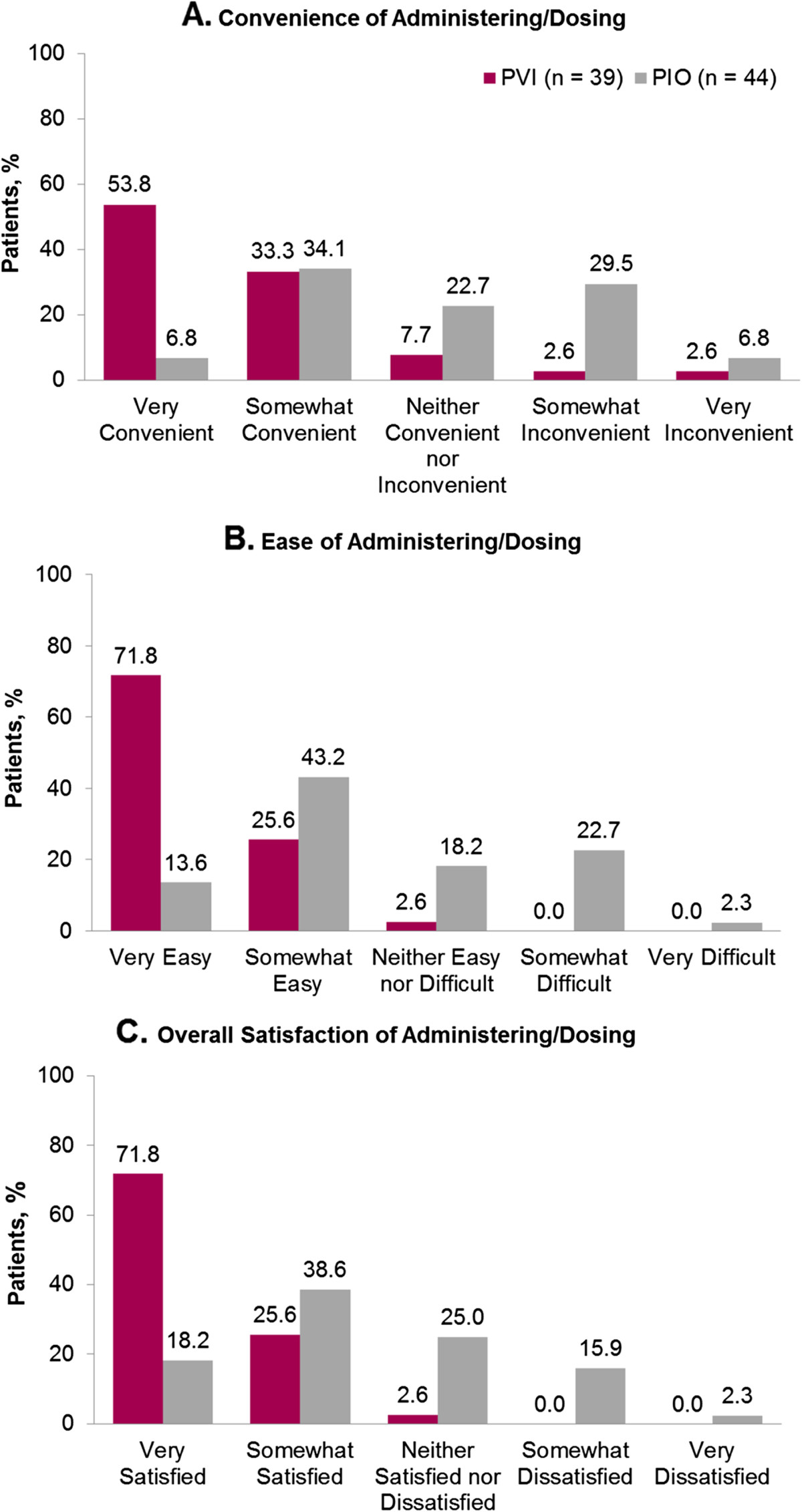
**Convenience, ease, and overall patient satisfaction of administering/dosing progesterone vaginal insert (PVI) and progesterone in oil (PIO).**

The convenience/ease of administration and overall patient satisfaction for PVI or PIO based on survey responses at the final study visit are shown in Figure 1. More women commented on the convenience and ease of administration of PVI than IM PIO. Conversely, a majority of patients and their partners described pain and anxiety, respectively, associated with PIO. Progesterone treatment was reported as “very” or “somewhat convenient” to administer by 87.1% in the PVI group compared with 40.9% in the PIO group (Figure 1A). The “ease of use” of progesterone treatment was considered “very” or “somewhat easy” to administer by 97.4% in the PVI group compared with only 56.8% in the PIO group (Figure 1B). In the PIO group, 65.9% found treatment “very” or “somewhat painful”. Overall satisfaction with progesterone treatment was higher for PVI compared to PIO (71.8% of women were “very satisfied” with PVI compared with only 18.2% of women who received PIO) (Figure 1C).In the study, 24% (11/46) and 31% (15/49) of patients in the PVI and IM PIO arms, respectively, had used progesterone in a previous treatment cycle. Of these, 90.9% in the PVI group found vaginal inserts “very” or “somewhat easier” to use than a previous progesterone treatment. For those women in the PIO group, only 33.3% found this treatment “very” or “somewhat easier” to use than any previous progesterone treatment. In addition, 47.7% of patients’ partners in the PIO group reported being “very” or “somewhat anxious” about administering the treatment. No women in the PVI group found the vaginal inserts “difficult to administer” or expressed “dissatisfaction with administration” of the insert (Figure 1B and C). Overall, more patients expressed a preference for PVI than IM PIO.

### Pregnancy rates

There were no significant differences in the biochemical, clinical, and ongoing pregnancy rates for those patients in the PVI group compared with those rates for patients in the PIO group (see Table 2). Biochemical pregnancy rates were 60.4% and 64.9% for the PVI and PIO groups, respectively (*P* = 0.853). Clinical pregnancies occurred in 50.9% in both the PVI and PIO groups (*P* = 1.0). The ongoing pregnancy rates were 47.2% and 49.1% for the PVI and PIO groups, respectively (*P* = 0.851).

### Adverse events

One or more AEs were reported during progesterone treatment by 30.2% in the PVI group and 31.6% in the PIO group. Most AEs were of mild or moderate intensity in both groups. Abdominal distension, lower abdominal pain, nausea, and ovarian hyperstimulation syndrome (OHSS) were the most common AEs reported in both groups.

## Discussion

The IM and vaginal routes of administration are used almost exclusively in clinical practice when progesterone is given to patients for luteal phase support during ART [4, 6]. Reports indicate that the vaginal and IM administration of progesterone results in comparable pregnancy outcomes [6, 13, 15].

Moreover, this analysis provides a comprehensive, prospective comparison of patient-reported convenience and ease of use, overall patient satisfaction, and tolerability for PVI compared to IM PIO from a population of women undergoing cycles of IVF. The most distinguishing difference between PVI and PIO in this study was the overall patient preference for PVI. Despite the need to administer PVI either twice a day or 3 times a day, 82.6% of the patients in the PVI group found it “very” or “somewhat convenient” compared with 44.9% of women in the PIO group. Furthermore, 90% of those in the PVI group found this treatment to be “very” or “somewhat easier” to use than a previous progesterone treatment. This is in contrast to patient response in the PIO group, where 33.3% found PIO “very” or “somewhat easier” to use than a previous progesterone treatment.

Only a few studies with progesterone for luteal phase support during cycles of IVF have attempted to assess patient treatment satisfaction and ease of use. In two previous studies comparing administration of progesterone vaginal gel to IM PIO, patients reported better satisfaction with vaginal gel when compared to IM PIO [15, 18]. In a study by Schoolcraft et al., patients found the vaginal gel easier to use, less painful, and less time consuming to administer when compared to treatment with IM PIO during a previous cycle of IVF [19]. Data on patient satisfaction were obtained prospectively in these studies; however, the data collected were more limited in scope than the data collected in the current study. Results from this survey of patient satisfaction, convenience, ease of use, and tolerability provide a more comprehensive assessment of patient-reported satisfaction than analysis included in previous studies.

No significant difference in biochemical, clinical, or ongoing pregnancy rates were observed among the treatment groups. Pregnancy outcomes in this study were similar to those reported in previous studies comparing vaginal to IM administration of progesterone for luteal support in women of a similar age range [6–11, 14, 15].

Progesterone administered vaginally for luteal phase support has been shown to be well tolerated. The most common treatment-emergent AEs associated with PVI include nausea, abdominal pain, OHSS, and oocyte retrieval pain [22]. Breast tenderness, headache, abdominal pain, abdominal distension, nonspecific muscle spasms, and vaginal discharge have been associated with vaginal gel [23].

It can be difficult to clearly elucidate the true cause-effect relationship of AEs reported during a clinical study. Nausea, OHSS, abdominal distention, and abdominal pain reported most frequently during this study by both PVI and PIO users were also commonly reported AEs when medications given for ovarian stimulation and IVF were used [21, 22, 24, 25]. Injection site irritation and redness have been reported frequently with administration of IM PIO [18, 26].

The results of this comprehensive, prospective patient survey and other similar reports suggest that PVI provides an easy-to-use and convenient method for providing the necessary luteal phase support for IVF cycles without the pain and inconvenience of daily IM injections and with comparable pregnancy rates.

## Authors’ original submitted files for images

Below are the links to the authors’ original submitted files for images. Authors’ original file for figure 1 Authors’ original file for figure 2

## Abbreviations

AE: Adverse event
ART: Assisted reproductive technology
BMI: Body mass index
E_2_: Serum estradiol
FSH: Follicle-stimulating hormone
GnRH: Gonadotropin-releasing hormone
GnRHa: Gonadotropin-releasing hormone agonist
hCG: Human chorionic gonadotropin
HP-hMG: Highly purified human menopausal gonadotropin
IM: Intramuscular
IVF: In vitro fertilization IVF
OHSS: Ovarian hyperstimulation syndrome
PCOS: Polycystic ovarian syndrome
PIO: Progesterone in oil
PVI: Progesterone vaginal insert
rFSH: Recombinant FSH
SC: Subcutaneously
TVU: Transvaginal ultrasonography.
