# Supplementary figures and images for: Patients’ administration preferences: progesterone vaginal insert (Endometrin®) compared to intramuscular progesterone for Luteal phase support
Source: Reprod Health. 2014 Nov 11;11:78. doi: 10.1186/1742-4755-11-78 (PMC4414383; doi:10.1186/1742-4755-11-78)

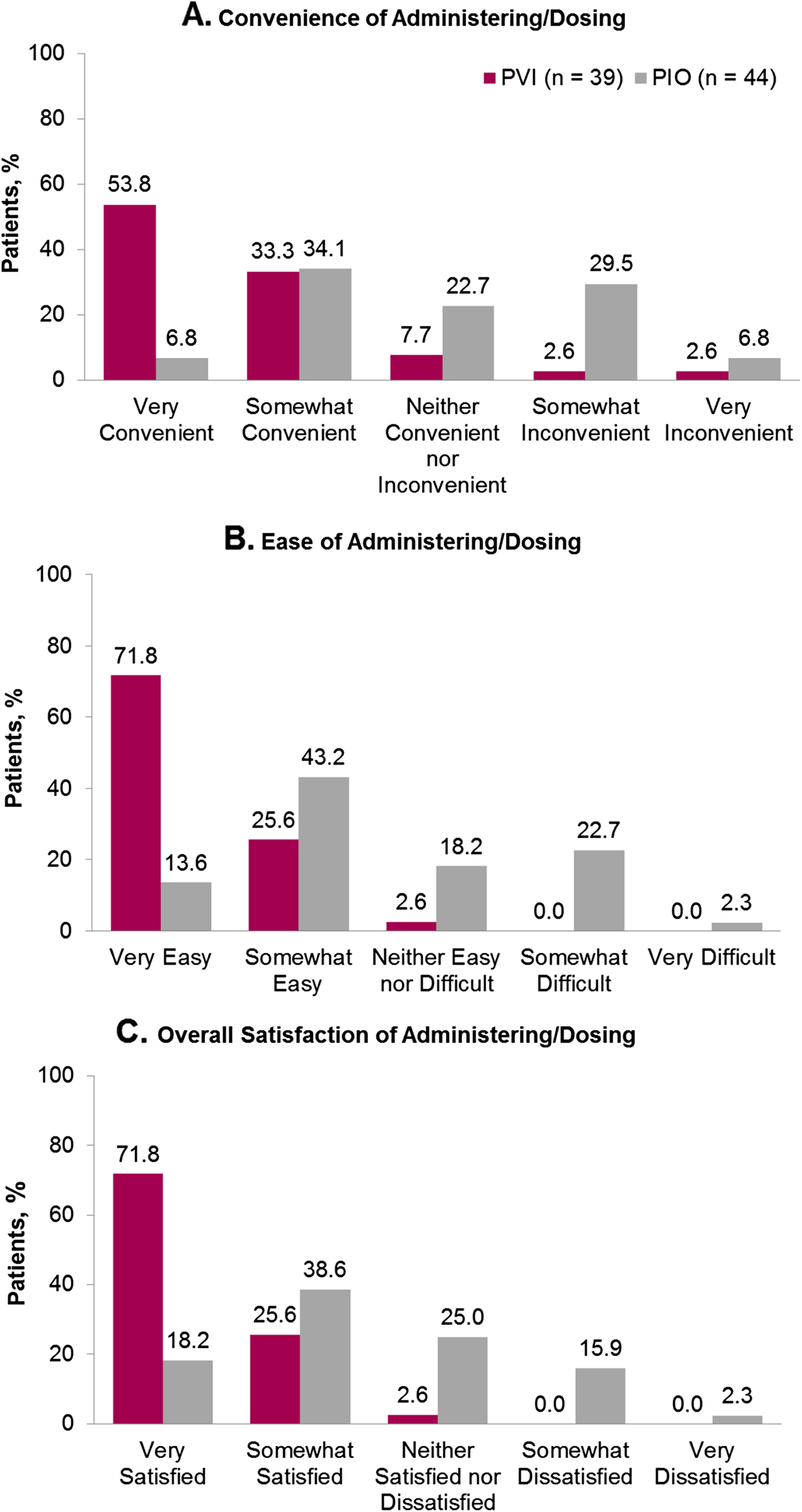

Supplement: Supplementary file 1 — Authors’ original file for figure 1 [file 12978_2013_362_MOESM1_ESM.tiff]

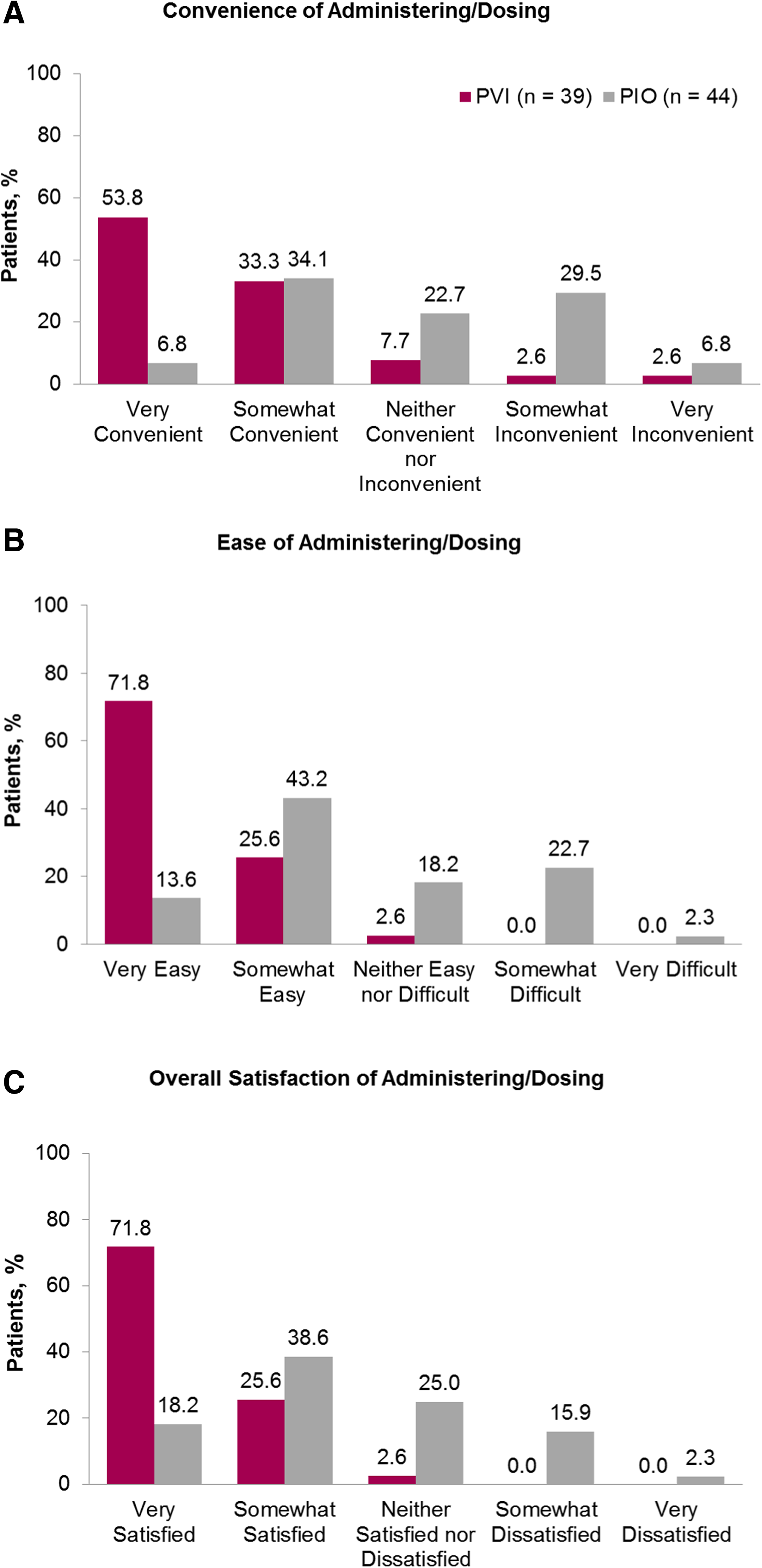

Supplement: Supplementary file 2 — Authors’ original file for figure 2 [file 12978_2013_362_MOESM2_ESM.tif]
